# Regional variation in biomechanical properties of ascending thoracic aortic aneurysms

**DOI:** 10.1093/ejcts/ezac392

**Published:** 2022-07-27

**Authors:** M Yousuf Salmasi, Sumesh Sasidharan, Jennifer Frattolin, Lowell Edgar, Ulrich Stock, Thanos Athanasiou, James Moore Jr

**Affiliations:** Department of Surgery and Cancer, Imperial College London, London, UK; Department of Bioengineering, Faculty of Engineering, Imperial College London, London, UK; Department of Bioengineering, Faculty of Engineering, Imperial College London, London, UK; Department of Bioengineering, Faculty of Engineering, Imperial College London, London, UK; Department of Cardiac Surgery and Transplantation, Royal Brompton and Harefield Foundation Trust, London, UK; Department of Surgery and Cancer, Imperial College London, London, UK; Department of Bioengineering, Faculty of Engineering, Imperial College London, London, UK

**Keywords:** Thoracic aortic aneurysms, Uniaxial tensile test, Delamination test, Material properties, Biomechanics

## Abstract

**OBJECTIVES:**

This study aims to characterize the material properties of ascending thoracic aortic aneurysmal tissue, using regional biomechanical assessment of both tensile and dissection propagation peel strength.

**METHODS:**

Thirty-four aneurysm specimens (proximal thoracic aorta) were harvested en-bloc from patients undergoing surgery for aneurysm replacement. Specimens were processed into regional samples of similar shapes covering the whole aneurysm isosurface, according to a structured protocol, in both orientations (longitudinal and circumferential). Thickness mapping, uniaxial tensile and peel tests were conducted, enabling calculation of the following parameters: true stress/strain, tangential modulus, tensile strength, peeling force and dissection energy. Two constitutive material models were used (hyperelastic models of Delfino and Ogden) to fit the data. A circumferential strip of tissue was also obtained for computational histology [regional quantification of (i) elastin, (ii) collagen and (iii) smooth muscle cells].

**RESULTS:**

The aortic wall was thinner on the outer curve (2.21, standard deviation (SD) 0.4 mm vs inner curve 2.50, SD 0.12 mm). Advanced patient age and higher pulse wave velocity (externally measured) were predictors of increased aortic wall thickness. Tensile strength was higher in the circumferential versus longitudinal direction when analysed according to anatomical regions. Both peel force (35.5, 22 N/m) and dissection energy (88.5, 69 J/m^2^) were on average lowest at the outer curve of the aneurysm in the longitudinal orientation. Delfino and Ogden model constants varied throughout anatomical regions, with the outer curve being associated a higher ɑ constant (Delfino) and lower µ1 constant (Ogden) (*P* < 0.05) indicating increased stiffness. Histologically, collagen abundance was significantly related to circumferential and longitudinal strength (*P*= 0.010), whilst smooth muscle cell count had no relation with any mechanical property (*P* > 0.05).

**CONCLUSIONS:**

Our results suggest that the outer aortic curve is more prone to dissection propagation and perhaps less prone to rupture than the inner aortic curve. This strengthens the notion of disease heterogeneity in ascending thoracic aortic aneurysms and has implications for the pathogenesis of aortic dissection.

**Figure:**
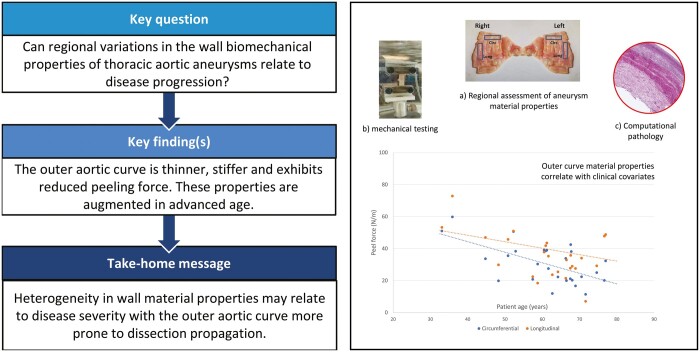

## INTRODUCTION

The unmet clinical need in the management of ascending thoracic aortic aneurysms (ATAA) is the lack of an accurate predictive model or biomarker for the risk of acute aortic syndrome [1, 2], Current guidelines rely only on aneurysm size and growth to determine the risk of type A aortic dissection (TAAD) [3]. These guidelines provide only limited guidance to clinicians in selecting patients for surgical intervention alongside considerations of disease subgroups, such as connective tissue disease and bicuspid aortopathy.

Size criteria is a blunt tool and relies heavily on Laplace’s law, oversimplifying the complexity of the disease to a single surrogate measure for aortic wall tension, thus discounting local alterations in wall stress, non-cylindrical geometries and material properties. Indeed, TAAD has been shown to arise in up to 40–60% cases where the aorta is below the size threshold [4, 5], meaning that guidelines may often miss at-risk patients.

Determining these local variations in aortic wall mechanics, although currently not possible using non-invasive/imaging techniques, may aid in achieving patient-specific characterization of ATAA and more accurate prognostication. *In vitro* testing of explanted tissue has unlocked the potential for such characterization. Although not all TAAD occurs in the context of aneurysms, the analysis of excised ATAA specimens can provide invaluable insights into mechanisms for aortic wall tissue failure. Several studies have observed both non-linear elasticity and anisotropy in aortic wall specimens [6–10] and data can be incorporated into biomechanical models to estimate stresses and establish links to pathologic patterns. However, few studies have searched for regional variation within aneurysms [11]. These efforts have been limited by specimen numbers, optimization of regional sampling, absence of thickness data and/or the absence of linked tensile and delamination testing.

We have gained access to surgically resected ascending aortic tissue specimens and undertook the task of characterizing their mechanical and histological properties. We compared the outcomes of these measurements to non-invasive clinical measures that could be gathered from any patient to guide surgical strategies.

## METHODS

We performed a prospective multicentre cohort study of patients undergoing surgery for proximal aortic aneurysms who were recruited consecutively during February 2018 and December 2019 from 4, specialized, aortic surgical centres.

### Ethics statement

The study received ethical approval from the Health Research Authority and Regional Ethics Committee (17/NI/0160, August 2017) and was sponsored by the Imperial College London Joint Research and Compliance Office, as defined under the sponsorship requirements of the Research Governance Framework (2005). All subjects provided written informed consent and their participation in the research study did not cause any deviation from routine clinical practice or additional diagnostic/therapeutic procedures.

### Inclusion and exclusion criteria

Thirty-four patients undergoing elective surgical replacement of an aneurysmatic ascending aorta and/or root were recruited (i.e. aneurysm >5.5 cm diameter or >5 cm and requiring intervention on the aortic valve) (Supplementary Material, Appendix S1). To ensure a homogenous cohort, patients with known connective tissue disease (i.e. Marfans, Ehler–Danlos, Loeys–Dietz), bicuspid aortic valves and emergency dissection cases were excluded. To ensure consistency in the quality of obtained aortic tissue, patients undergoing redo sternotomy were excluded.

### Aortic tissue acquisition

**Figure 1:**
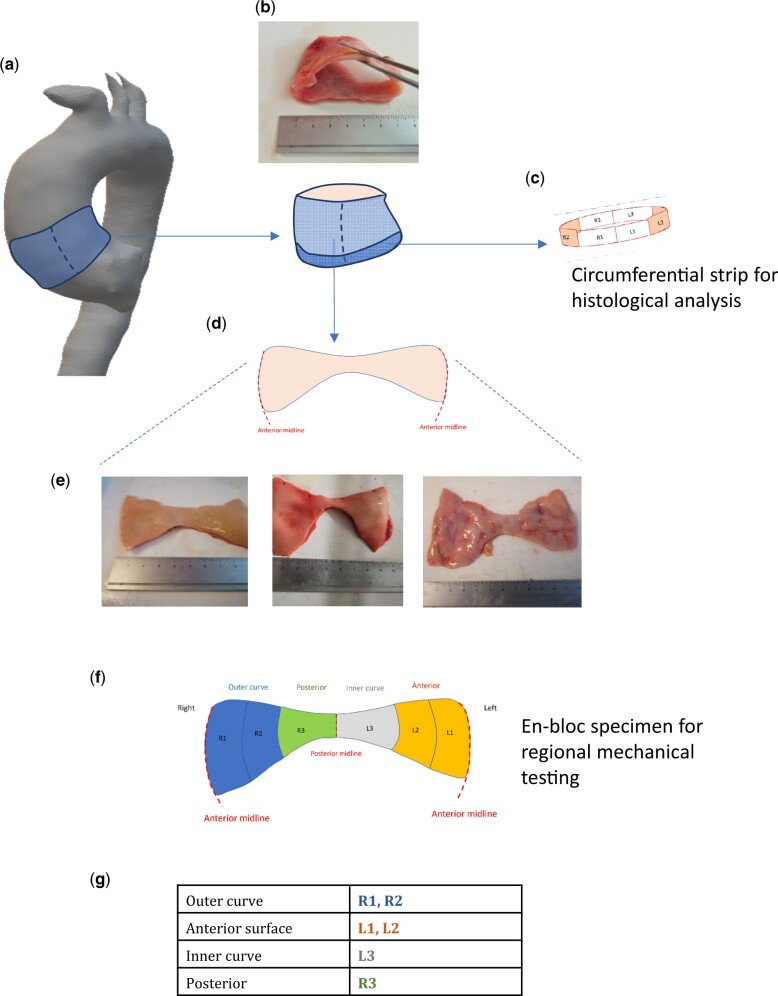
Origin of the ascending thoracic aortic aneurysm specimen and estimated anatomical regions. The proximal ascending thoracic aortic aneurysm specimen is excised en-bloc (**A** and **B**). From the inferior-most border, a 4–5-mm strip of tissue is removed and processed for histological analysis ©. The remainder of the specimen is divided vertically down the anterior midline giving the characteristic butterfly appearance (**D**). Photographs of actual patient specimens are shown in E (intimal surface facing up). The circumferential regions for subsequent analysis are 6 equal regions (**F**), corresponding to the anatomical locations described in the table (**G**).

Thirty-four aortic specimens were successfully obtained during surgery and subjected to mechanical characterization. The distribution of aneurysm pathology was: 12 root, 13 ascending, 5 root and ascending and 4 ascending and arch. Aneurysm specimens were obtained en-bloc and acquired immediately after surgical excision in the operating theatre. A mark made on the antero-superior aspect of the aortic tissue allowed for the precise orientation of the specimen (Fig. 1) (Supplementary Material, Appendix S2). The whole aortic specimen was immersed in a 10% dimethyl sulphoxide solution and stored at −80°C [12, 13]. Prior to this, a circumferential strip (4–5-mm axial extent) of the whole ATAA specimen was obtained from its inferior-most border, immersed in formalin and used for regional histological analysis (including quantification of elastin, collagen and smooth muscle cell count—Supplementary Material, Appendix S3).

### Sample preparation

The term ‘sample’ will be used henceforth to denote a segment derived from the whole ATAA specimen collected from each patient. Frozen aortic tissue specimens were defrosted at 4°C for 24 h. After thawing, the specimens were vertically divided on the anterior wider aspect, laying open in a ‘butterfly’ shape (Fig. 1). The thickness of specific regions of the specimen was determined utilizing a Litematic VL-50-B measuring device (Mitutoyo Ltd.), with a precision of 0.1 µm.

**Figure 2:**
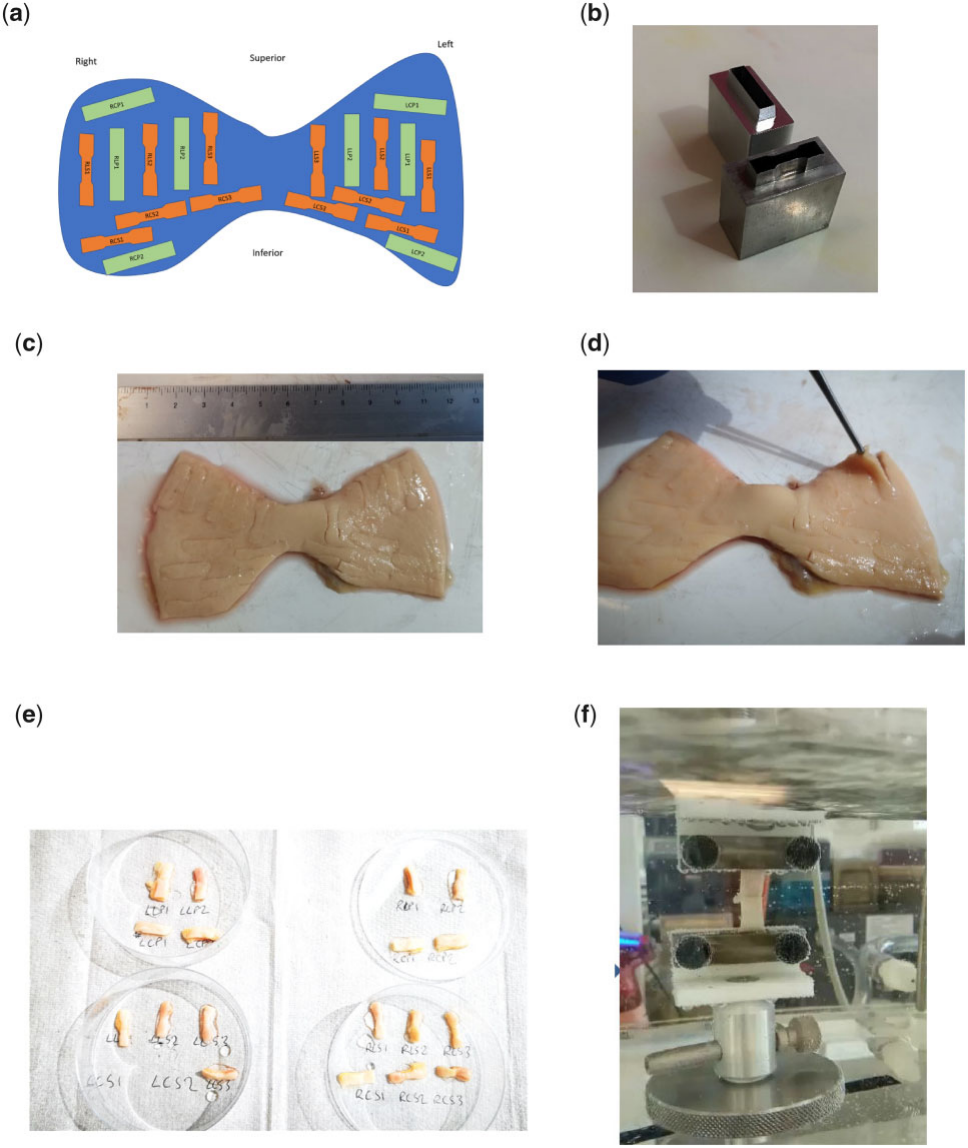
Processing ATAA specimens to obtain tensile and peel testing samples. (**A**) protocol for obtaining the different samples throughout the whole specimen as a schematic diagram including both the dogbone samples (orange) for mechanical property assessment and rectangular samples (green) for peel tests. Image (**B**) shows the metallic stencils used to cut out the testing samples (both dogbone and rectangular shaped) measuring at 5 mm × 20 mm. Images (**C**) and (**D**) show 2 different ATAA specimens in which the samples have been punched out and ready for dissection with a scalpel. Ima©(**E**) shows the excised samples labelled and immersed in phosphate-buffered saline, whilst (**F**) shows a uniaxial tensile test in progress in the environmental chambre.

Due consideration of the aortic anatomy was made to identify its various regions (Supplementary Material, Appendix S2). For uniaxial tensile testing, a sharp stencil was used to create 20 mm × 5 mm dogbone samples (with gauge length of 10 mm, width of 4 mm), in both the circumferential and longitudinal orientations. Six circumferential samples were obtained at the lowermost end of the specimen covering the entire aneurysm from left to right, followed by 6 longitudinal samples from the middle aspect of the aneurysm specimen (Fig. 2). For peel testing, a rectangular stencil was used to obtain 8 samples measuring 20 mm × 5 mm from both halves of the aneurysm specimen, as well in the circumferential and longitudinal directions (Fig. 2). The resulting 20 samples were immersed in phosphate-buffered saline and kept at room temperature prior to mechanical testing.

### Uniaxial tensile testing

**Figure 3:**
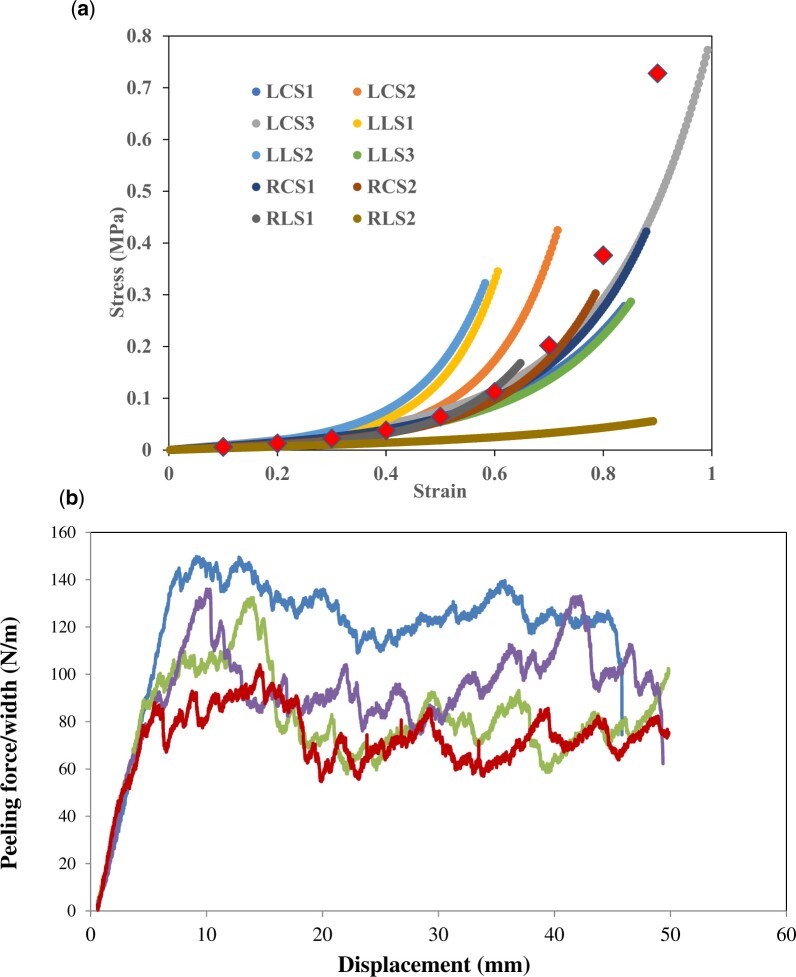
(**A**) Uniaxial raw stress–strain data from 12 dogbone samples obtained from a single ATAA specimen. Each plotted line represents a different aortic wall sample, varying in anatomical location and orientation (longitudinal versus circumferential). (**B**) Graphical representation of peeling experiments for 4 separate ATAA samples. The applied peeling test causes a slow and controlled dissection propagation, where the force–displacement curve is characterized by a jagged plateau region.

Uniaxial tensile measurements of extension force and elongation were performed using a Test Resources R-Series Controller with a 44-N load cell. All tests were conducted in an environmental chamber containing phosphate-buffered saline, maintained at 37°C. The dogbone samples were individually mounted lengthwise between serrated clamps, following recommended designs for soft tissue clamping [14]. Samples were preconditioned using published soft tissue experimental protocols with a preset controlled force of 0.01 N at 2 mm/min (between 0% and 20% strain) and repeated for 5 cycles [15, 16]. The sample was then stretched at a constant crosshead speed of 2 mm/min, until sample rupture (Fig. 3A).

Cauchy stress ($\sigma_{T}$), in MPa, was calculated according to the following equation:

$$\sigma_{T}=\frac{F}{A_{o}}\left(1+\varepsilon \right)$$

where *F* is the load in N, $A_{o}$ is the original cross-sectional area in mm^2^ and ε is the strain defined as

$$\varepsilon =\frac{\Delta l}{l_{o}}$$

where Δl is the change in length at any instant in mm and $l_{o}$ is the original gauge length in mm.

From the resulting stress–strain curve, the ultimate tensile strength (UTS), in MPa, was determined.

### Fitting data to constitutive models

Both the Ogden and Delfino hyperelastic material models were fitted to the generated force–elongation data, along with geometric measures, to estimate material constitutive parameters in an appropriate nonlinear finite deformation framework [15]. Briefly, the strain energy density function according to the Ogden model is defined as

$$\bar{\psi }=\;\sum_{i\;=\;1}^{N}\frac{{µ}_{i}}{\alpha_{i}}\;({{\bar{\lambda }}_{1}}^{\alpha_{i}}+\;{{\bar{\lambda }}_{2}}^{\alpha_{i}}\;+\;{{\bar{\lambda }}_{3}}^{\alpha_{i}}–\;3)$$

where *N* is the order of the model (usually between 1 and 3), µ_i_ and α_i_ are empirically derived material constants and λ_1_, λ_2_ and λ_3_ are the principal stretches.

The Delfino model is described by the following equation:

$$\bar{\psi }=\frac{a}{b}\left\{\exp\left[\frac{b}{2}\;({\bar{I}}_{1}–3)\;\right]–\;1\right\}$$

where *a* and *b* are empirically derived material constants and ${\bar{I}}_{1}$is the Cauchy–Green tensor.

Fitting was performed with the non-linear regression software Hyperfit (v. 1.169, Brno, Czech Republic). To determine the optimal values of the material constants, the coefficient of determination, *R*^2^ and root mean square error were used to assess the resulting fits.

#### Delamination testing

An incision was made within the aortic media to create 2 flaps of tissue of 5–10 mm in length. Each flap was mounted onto serrated clamps, ensuring the inflection point of the peel was equidistant from both clamps. The load was applied perpendicularly to the incision plane, simulating aortic dissection. The sample was peeled at a rate of 10 mm/min [17], until complete separation of the sample. The peeling force was normalized to the sample width and expressed in N/m. The mean peeling force was calculated from the plateau region of the resulting force versus displacement curve, whilst dissection energy was calculated based on the area under the force versus displacement curve (Fig. 3B) (Supplementary Material, Appendix S4).

### Medical data

Demographic data, anthropometric measurements and clinical/radiological data were obtained via access to clinical records with patient consent. This enabled the comparison of ATAA wall material properties with clinically obtainable information. An additional (preoperative) non-invasive measurement of central aortic pressure and pulse wave velocity (PWV) was obtained using a combination of brachial cuff, femoral cuff and carotid artery probe application, connected to a purpose-built device with a specialized algorithm (Sphygmacor, AtCor Medical, Sydney, Australia).

### Statistical analysis

Sample size estimation was based on the predicted regional difference in mechanical properties of the ATAA wall, up to 150 N/cm^2^ (peak elastic modulus) [6]. To detect a regional difference with 90% power at a significance level of 0.05, the minimum sample size is 20 subjects.

From 34 ATAA specimens, a total of 354 individual samples successfully underwent thickness measurement and uniaxial tensile testing, whilst 208 samples underwent delamination testing. Values arising from each tested aortic segment were considered as separate data points. Continuous data were reported as means and standard deviations, averaged across all patients per region. Normality in data distribution was confirmed using the Shapiro–Wilk test.

Analysis of varianvce (ANOVA) was used to test the influence of region and orientation on the derived UTS values. The data were then modelled into a clustered hierarchical structure, nested into each patient as a separate cluster, followed by the orientation of the segment (circumferential versus longitudinal). This allowed for multilevel mixed-effect linear regression models to be constructed analysing the effect of ATAA region on material properties of the aortic wall.

Material properties tested against clinical covariates [age, height, body mass index (BMI), mean arterial pressure, etc.] using univariate linear regression analysis. Results of these analyses were reported as standardized beta coefficients with 95% confidence intervals (CIs). Region-specific elastin/collagen abundance (derived from computational pathology) was also compared to mechanical (tensile and peel) data using linear regression analysis.

Subgroup analysis was conducted to compare wall material properties of the inner curve with properties of the outer curve. Aneurysm dimension (namely maximal diameter) was also tested against mechanical properties. The significance level for all models was set at α = 0.05. All statistical analysis was conducted using STATA 13.0 (Stata Corp., College Station, TX, USA).

## RESULTS

### Aortic wall thickness

**Figure 4:**
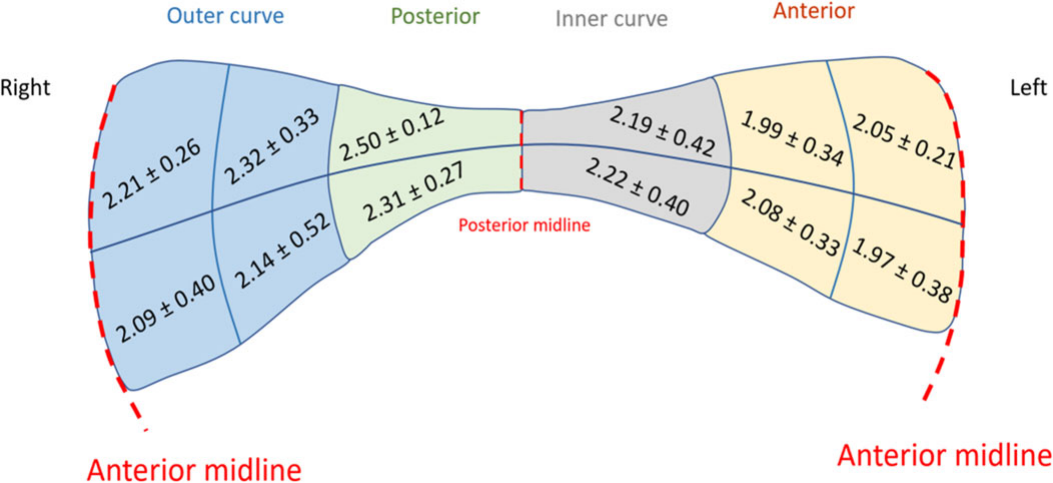
Average thickness (mm) according to ascending thoracic aortic aneurysm anatomical region. Values given are mean and standard deviations according to the pre-defined regions in Fig. 1. Each region also had a superior and inferior aspect, thus giving 12 regions of derived thickness values.

Tissue thickness ranged from 1.5 to 3.5 mm, exhibiting a normal distribution. The posterior aortic wall was on average thicker than the anterior and outer curve regions [2.50 (0.12) vs 1.97 (0.38) mm, paired sample *t*-tests *P* = 0.0066 and *P* = 0.0490, respectively] (Fig. 4 and Supplementary Material, Appendix S5).

### Tensile test data

Analysis of average UTS values revealed higher values at the posterior wall (1.002 (0.543) MPa) and lowest values at the inner curve (0.591 (0.509) MPa) (Supplementary Material, Appendix S6). Given the normally distributed data, ANOVA testing was conducted to assess the influence of tissue orientation (longitudinal versus circumferential) and tissue sample location (inner versus outer curve) on ATAA wall mechanical properties, finding orientation to have a more significant influence on UTS (*P* = 0.0001) than location (inner versus outer), indicating that these aortic walls would be more likely to fail in the longitudinal direction than the circumferential direction, regardless of location (Supplementary Material, Appendix S7). Assuming a linear relationship with the data, at a longitudinal UTS of 0.75 MPa, the UTS in the circumferential direction is more than double (2.0 MPa) (Supplementary Material, Appendix S8).

### Constitutive models

Stress–strain data were fitted to both the Delfino and Ogden constitutive equations. Fitted data provided values of coefficient of determination all greater than 0.95, thus suggesting a satisfactory fitting of uniaxial stress–strain profiles (Supplementary Material, Appendix S9). Each sample yielded 2 and 4 material constant values for the Delfino and Ogden models, respectively. Multilevel mixed-effect linear regression models to be constructed analysing the effect of ATAA region on material properties of the aortic wall, accounting for data clustering in the patient domain and sample orientation (Table 1). Aortic tissue from the outer curvature was associated with higher values of the ɑ-constant from the Delfino model (coef 0.014, 95% CI [0.0036–0.0249], *P* = 0.009) and smaller (and more negative values) of the µ1-constant of the Ogden model (coef −0.995, 95% CI [−1.868, −0.121], *P* = 0.009) (Table 1).

**Table 1:** Results of multilevel mixed-effects linear model to assess the influence of anatomical region on the material constants of the aortic wall in ATAA

	Coef	Standard error	95% CI	*P*-Value
(a)				
Outer curve versus other regions	0.014	0.005	0.0036–0.0249	0.009
Patient	0.032	0.019	0.010 to 0.105	
Orientation	5.13 × 10^−24^	–	–	
Var (estimate ɑ-Delfino parameter)	0.002	0.0019	0.0017 to 0.0025	
Likelihood ratio test versus linear model				0.027
(b)				
Outer curve versus other regions	−0.995	0.446	−1.868 to −0.121	0.009
Patient	0.512	0.499	0.076 to 3.459	
Orientation	3.17 × 10^−18^	–	–	
Var (estimate µ1-Ogden parameter)	5.937	0.891	4.424–7.967	
Likelihood ratio test versus linear model				0.428

The fixed effects part of the model tested the influence of anatomical region (outer curve versus other regions) on the (a) ɑ-Delfino parameter and (b) µ1-Ogden parameter. The random effect part of the models tested the influence of the patient, and sample orientation (longitudinal versus circumferential). From these results, the influence of region on the material property is significant, with the added influence of the patient from which the sample was sourced. The effect of sample orientation on the value of the material constant in these models was negligible.

CI: confidence interval.

### Delamination of ATAA tissue

The average peeling force was found to be lower in the circumferential direction compared to the longitudinal direction in all regions. The data also showed lowest values for peeling force in the outer curve, which was more apparent in longitudinal samples (paired sample *t*-test *P* < 0.05). These trends were also reflected in the dissection energy data (Supplementary Material, Appendix S10). ANOVA found sample orientation to have a significant influence on peel force (*P* = 0.0061) more so than sample location (Supplementary Material, Appendix S7).

### Influence of clinical factors on mechanical properties

**Figure 5:**
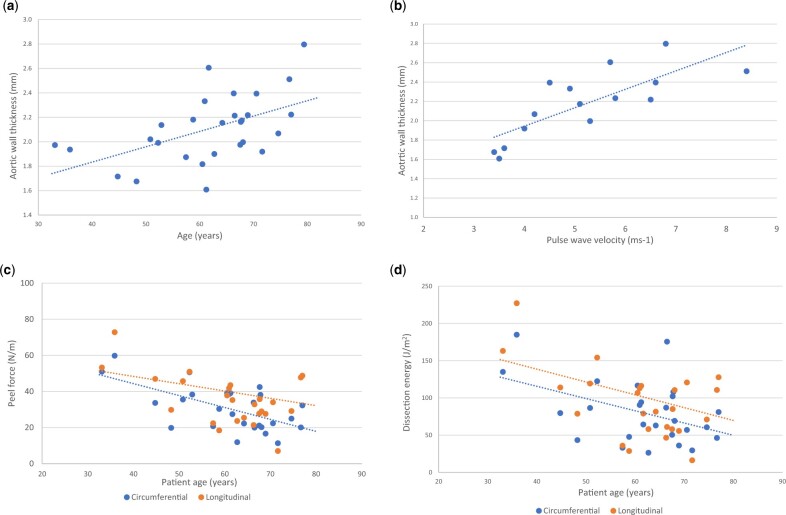
Scatter plots displaying the relationship between patient factors patient-specific ascending thoracic aortic aneurysm material properties. There is a significantly positive relationship between aortic wall thickness and patient age (**A**) and pulse wave velocity (**B**). Plots (**C**) and (**D**) display the relationship between patient age peel force (**C**) and dissection energy (**D**). Both these plots show that increasing age has a significant negative effect on peel force/dissection energy, and thus higher risk of aortic dissection.

Regression analysis comparing tissue thickness with clinical covariates revealed patient age (standardized β coef 0.0125, 95% CI [0.0044, 0.0207], *P* = 0.004) and PWV (*P* = 0.007) as positive predictors of increased wall thickness (Fig. 5 and Table 2), whilst other covariates (height, BMI, mean arterial pressure and maximum aneurysm diameter) were not significantly related (Supplementary Material, Appendix S12).

**Table 2:** Linear regression analysis: association between patient covariates and ATAA material properties (wall thickness, tensile/peel properties) (complete table in Supplementary Material, Appendix S11)

Covariate	Coef	Standard error	95% CI	*P*-Value
Tissue thickness				
Patient age	0.0125	0.004	0.0044 to 0.0207	**0.004**
Max aneurysm diameter	0.0152	0.010	−0.0056 to 0.0361	0.139
Pulse wave velocity	0.1728	0.050	0.0595 to 0.2861	**0.007**
Smoking	0.2022	0.138	−0.0793 to 0.4837	0.153
Ultimate tensile strength—longitudinal				
Patient age	−0.0097	0.003	−0.0160 to −0.0035	**0.004**
Max aneurysm diameter	−0.0141	0.008	−0.0315 to 0.0034	0.105
Pulse wave velocity	−0.0761	0.026	−0.1353 to −0.0168	**0.017**
Smoking	0.0671	0.094	−0.127 to 0.261	0.484
Peel force—longitudinal				
Patient age	−0.404	0.443	−1.318 to 0.510	0.371
Max aneurysm diameter	−0.506	1.190	−3.077 to 2.065	0.678
Pulse wave velocity	−1.189	7.298	−17.699 to 15.321	0.874
Smoking	13.160	11.152	−9.764 to 36.084	0.249
Dissection energy—circumferential				
Patient age	−1.642	0.669	−3.022 to −0.262	**0.022**
Max aneurysm diameter	−1.948	1.491	−5.170 to 1.274	0.214
Pulse wave velocity	−4.648	4.846	−15.612 to 6.315	0.363
Smoking	1.885	18.868	−36.899 to 40.669	0.921

Bold type indicates statistical significance.

When comparing UTS, age and PWV displayed a consistent negative relationship with wall strength in both longitudinal and circumferential orientations. This is to say that advancing age and higher PWV values were both associated with lower UTS values. Interestingly, maximum ATAA diameter did not influence strength or peel force for either orientation, and neither did patient height nor BMI (*P* > 0.05) (Supplementary Material, Appendix S12). The only covariate found to significantly influence peel force and dissection energy was patient age, and this was only the case for the circumferential delamination (peel force: coef −0.660, 95% CI [−1.016, −0.305], *P* = 0.001 and dissection energy: coef −1.642, 95% CI [−3.022, −0.262], *P* = 0.002) (Fig. 5 and Table 2).

### Relationship between mechanical properties and histology

Collagen abundance displayed a significantly positive relationship with UTS in both the circumferential and longitudinal directions (Supplementary Material, Appendix S13). Collagen abundance was found to significantly increase both the peel force and dissection energy in the circumferential direction, as well as the dissection energy in the longitudinal direction (*P* > 0.05). This was a positive relationship, indicating that a higher collagen abundance leads to a higher peel force/dissection energy value. Elastin content and VSMC count were found to have no relation with any of the mechanical properties (*P* > 0.05).

## DISCUSSION

Regional variability in wall stiffness and mechanical strength may contribute to the initiation of TAAD and subsequent aortic expansion during ATAA pathogenesis. Although some cases of TAAD can occur in non-aneurysmal aortas (up to 40% below size criteria), this study analysed patients with established aneurysmal disease as a model of TAAD predisposition. In mechanical terms, this models TAAD (in the context of ATAA) as a progressive tissue failure ensuing within a chronically altered aortic wall.

### Peel force and dissection energy 

The main failure mode of ATAA is separation of its medial layers, which are already degenerated in chronic aneurysmal disease. The incorporation of biomechanical models that relate closely to the relevant pathophysiology is crucial, hence the use of peel testing: characterizing the radial failure stress and energy required to propagate a dissection. Our data show for the first time the large variations in peeling force and dissection energy between adjacent areas in the ATAA wall (outer versus inner curve *P* = 0.0093). We have also identified a lower peel force in the outer curve region mainly in the longitudinal orientation, which is similar to previous smaller studies [18]. This may have important implications for the initiation of the entry tear in aortic dissection at particularly weak locations of the aortic wall. The circumferential orientation of entry tears has been minimally reported in the literature, whilst a considerable focus on tear site up the aortic progression (root, ascending, arch) is clinically relevant [19], as well as tear size [20].

### Wall thickness

Heterogeneity of wall thickness was a primary finding in ATAA. The ATAA wall was thinner anteriorly and on the outer curve and thicker posteriorly and on the inner curve. Thickness also displayed a significant positive relationship with age: older patients tended to have thicker aortas. This lays emphasis on the need for more accurate patient-specific modelling of the aortic wall, as opposed to assumptions of uniform wall thickness, in the context of computational studies [21–23]. Whilst it is possible that patients who exhibited thinner aortic walls were at higher risk of rupture, our surgical specimens were gathered from elective procedures prior to rupture. Previous studies of AAA disease found wall thickness to be the single main predictor of rupture potential, as well as inverse correlations with failure stress and direct correlations with peak elastic modulus [24].

PWV was found to be a better predictor of wall thickness than blood pressure with an increase in PWV by 5 m/s corresponding to an increase in wall thickness of 1 mm. PWV is increasingly being used in the clinical setting in the context of cardiovascular risk profiling. As far as we know, this is the first comparison of PWV to measurements of patient-specific aortic wall properties from explanted tissue.

### ATAA wall tensile properties

Increased stiffness in the circumferential direction, as demonstrated by our results, has been described by previous uniaxial studies on ATAA [25] and reinforces the anisotropic nature of the aortic wall. Our study also reinforces that regional variation in aneurysmatic degeneration region strongly influences the deformation properties of the aortic wall at larger strains. Specifically, the stiffer outer curve of the aorta was associated with a higher ɑ-Delfino value and a lower µ_1_-Ogden value; both these constants are stress/pressure-like constants representing the degree of non-linearity in the stress–strain curve. These findings challenge many biomechanical studies that assume a homogenous aortic wall structure with uniform thickness [17, 18] and have important implications on the generation of more accurate and patient-specific biomechanical models of aortic function, particularly in diseased states.

These findings can be explained by the altered composition of structural proteins (elastin/collagen) in the aortic media, as identified through regression analysis with matched histological samples. Reduced elastin, a hallmark of medial degeneration, leads to increased aortic stiffness, as does a predilection for increased collagen.

We also found no relationship between tensile strength and ATAA diameter which contrasts with the results of Azadani *et al.* [26], but their studies were performed using a biaxial tensile testing apparatus. In a more recent study by Duprey *et al.* [27], the use of bulge inflation testing (also biaxial) in 31 ATAA specimens yielded no relation with aneurysm diameter. However, numerous studies [28, 29] support the non-inferiority of uniaxial testing compared to biaxial and our data strongly support the assertion that aneurysm diameter is an inadequate surrogate for wall tensile strength.

### Limitations

Although the number of patients included in this study is roughly equivalent to other similar and important publications in this field, the cohort size is still somewhat limited. Our studies also provide data for ATAA patients with trileaflet aortic valves. There is no comparison with normal aortas (as a control group), neither is there a comparison with BAV aortas. Several studies have suggested the altered mechanical properties of BAV-related aneurysms, reporting increased stiffness and anisotropy compared to non-BAV ATAAs. We have recently noted [30] that clamp strain from tensile testing machines is not necessarily representative of the strain in the tissue. This is a systematic error of the nature that would not influence the comparative results presented here.

## CONCLUSION

This study has importantly highlighted the regional variation and anisotropy in the mechanical properties of the ATAA wall. This work has also demonstrated altered properties in the outer curve of the ATAA wall, namely a thinner wall with reduced peeling force. These features indicate increased susceptibility to aortic dissection. Furthermore, the relationships these parameters have with clinical variables highlight the interplay between patient and aortic phenotype beyond size criteria alone.

## SUPPLEMENTARY MATERIAL

Supplementary material is available at *EJCTS* online.

## Funding

This work was supported by the National Institute for Health Research (NIHR) Imperial College Biomedical Research Centre (P69559 and P74143 to Thanos Athanasiou).

**Conflict of interest:** none declared.

## Supplementary Material

ezac392_Supplementary_Data

## Data Availability

The data used in this manuscript can be made available upon request.

## ABBREVIATIONS

CIs: Confidence intervals
ATAA: Ascending thoracic aortic aneurysms
TAAD: Type A aortic dissection
UTS: Ultimate tensile strength
PWV: Pulse wave velocity
BMI: Body mass index

## References

[1] El-Hamamsy I , Yacoub MH. Cellular and molecular mechanisms of thoracic aortic aneurysms. Nat Rev Cardiol 2009;6:771–86. 19884902 10.1038/nrcardio.2009.191

[2] Braverman AC. Acute aortic dissection: clinician update. Circulation 2010;122:184–8. 20625143 10.1161/CIRCULATIONAHA.110.958975

[3] Erbel R , Aboyans V, Boileau C, Bossone E, Di Bartolomeo R, Eggebrecht H et al; ESC Committee for Practice Guidelines. 2014 ESC Guidelines on the diagnosis and treatment of aortic diseases: document covering acute and chronic aortic diseases of the thoracic and abdominal aorta of the adult. The Task Force for the Diagnosis and Treatment of Aortic Diseases of the European. Eur Heart J 2014;35:2873–926. 25173340 10.1093/eurheartj/ehu281

[4] Pape LA , Tsai TT, Isselbacher EM, Oh JK, O’Gara PT, Evangelista A et al Aortic diameter ≥5.5 cm is not a good predictor of type A aortic dissection: observations from the International Registry of Acute Aortic Dissection (IRAD). Circulation 2007;116:1120–7. 17709637 10.1161/CIRCULATIONAHA.107.702720

[5] Rylski B , Branchetti E, Bavaria JE, Vallabhajosyula P, Szeto WY, Milewski RK et al Modeling of predissection aortic size in acute type A dissection: more than 90% fail to meet the guidelines for elective ascending replacement. J Thorac Cardiovasc Surg 2014;148:944–8. 24998700 10.1016/j.jtcvs.2014.05.050

[6] Manopoulos C , Karathanasis I, Kouerinis I, Angouras DC, Lazaris A, Tsangaris S et al Identification of regional/layer differences in failure properties and thickness as important biomechanical factors responsible for the initiation of aortic dissections. J Biomech 2018;80:102–10. 30195853 10.1016/j.jbiomech.2018.08.024

[7] Smoljkić M , Fehervary H, Van den Bergh P, Jorge-Peñas A, Kluyskens L, Dymarkowski S et al Biomechanical characterization of ascending aortic aneurysms. Biomech Model Mechanobiol 2017;16:705–20. 27838784 10.1007/s10237-016-0848-4

[8] Phillippi JA , Pasta S, Vorp DA. Biomechanics and pathobiology of aortic aneurysms. In McGloughlin T (ed). Biomechanics and Mechanobiology of Aneurysms. New York, NY: Springer, 2011. pp. 67–118.

[9] Ferruzzi J , Vorp DA, Humphrey JD. On constitutive descriptors of the biaxial mechanical behaviour of human abdominal aorta and aneurysms. J R Soc Interface 2011;8:435–50. 20659928 10.1098/rsif.2010.0299 PMC3030820

[10] Roccabianca S , Ateshian GA, Humphrey JD. Biomechanical roles of medial pooling of glycosaminoglycans in thoracic aortic dissection. Biomech Model Mechanobiol 2014;13:13–25. 23494585 10.1007/s10237-013-0482-3 PMC3918738

[11] Di Giuseppe M , Alotta G, Agnese V, Bellavia D, Raffa GM, Vetri V et al Identification of circumferential regional heterogeneity of ascending thoracic aneurysmal aorta by biaxial mechanical testing. J Mol Cell Cardiol 2019;130:205–15. 30998978 10.1016/j.yjmcc.2019.04.010

[12] Bia D , Pessana F, Armentano R, Pérez H, Graf S, Zócalo Y et al Cryopreservation procedure does not modify human carotid homografts mechanical properties: an isobaric and dynamic analysis. Cell Tissue Bank 2006;7:183–94. 16933040 10.1007/s10561-005-0655-0

[13] Matsumoto T , Fukui T, Tanaka T, Ikuta N, Ohashi T, Kumagai K et al Biaxial tensile properties of thoracic aortic aneurysm tissues. J Biomech Sci Eng 2009;4:518–29.

[14] Jiang M , Lawson ZT, Erel V, Pervere S, Nan T, Robbins AB et al Clamping soft biologic tissues for uniaxial tensile testing: a brief survey of current methods and development of a novel clamping mechanism. J Mech Behav Biomed Mater 2020;103:103503. 32090940 10.1016/j.jmbbm.2019.103503

[15] Carew EO , Garg A, Barber JE, Vesely I. Stress relaxation preconditioning of porcine aortic valves. Ann Biomed Eng 2004;32:563–72. 15117030 10.1023/b:abme.0000019176.49650.19

[16] García-Herrera CM , Atienza JM, Rojo FJ, Claes E, Guinea GV, Celentano DJ et al Mechanical behaviour and rupture of normal and pathological human ascending aortic wall. Med Biol Eng Comput 2012;50:559–66. 22391945 10.1007/s11517-012-0876-x

[17] Noble C , Smulders N, Lewis R, Carré MJ, Franklin SE, MacNeil S et al Controlled peel testing of a model tissue for diseased aorta. J Biomech 2016;49:3667–75. 27743628 10.1016/j.jbiomech.2016.09.040

[18] Sommer G , Gasser TC, Regitnig P, Auer M, Holzapfel GA. Dissection properties of the human aortic media: an experimental study. J Biomech Eng 2008;130:021007. 18412494 10.1115/1.2898733

[19] Merkle J , Sabashnikov A, Deppe AC, Weber S, Mader N, Choi YH et al Impact of different aortic entry tear sites on early outcomes and long-term survival in patients with Stanford A acute aortic dissection. Thorac Cardiovasc Surg 2019;67:363–71. 29898464 10.1055/s-0038-1649511

[20] Evangelista A , Salas A, Ribera A, Ferreira-González I, Cuellar H, Pineda V et al Long-term outcome of aortic dissection with patent false lumen: predictive role of entry tear size and location. Circulation 2012;125:3133–41. 22615344 10.1161/CIRCULATIONAHA.111.090266

[21] Tan FPP , Borghi A, Mohiaddin RH, Wood NB, Thom S, Xu XY. Analysis of flow patterns in a patient-specific thoracic aortic aneurysm model. Comput Struct 2009;87:680–90.

[22] Wang X , Li X. Fluid-structure interaction based study on the physiological factors affecting the behaviors of stented and non-stented thoracic aortic aneurysms. J Biomech 2011;44:2177–84. 21722905 10.1016/j.jbiomech.2011.06.020

[23] Crosetto P , Reymond P, Deparis S, Kontaxakis D, Stergiopulos N, Quarteroni A. Fluid-structure interaction simulation of aortic blood flow. Comput Fluids 2011;43:46–57.

[24] Iliopoulos DC , Kritharis EP, Giagini AT, Papadodima SA, Sokolis DP. Ascending thoracic aortic aneurysms are associated with compositional remodeling and vessel stiffening but not weakening in age-matched subjects. J Thorac Cardiovasc Surg 2009;137:101–9. 19154911 10.1016/j.jtcvs.2008.07.023

[25] Iliopoulos DC , Deveja RP, Kritharis EP, Perrea D, Sionis GD, Toutouzas K et al Regional and directional variations in the mechanical properties of ascending thoracic aortic aneurysms. Med Eng Phys 2009;31:1–9. 18434231 10.1016/j.medengphy.2008.03.002

[26] Azadani AN , Chitsaz S, Mannion A, Mookhoek A, Wisneski A, Guccione JM et al Biomechanical properties of human ascending thoracic aortic aneurysms. Ann Thorac Surg 2013;96:50–8. 23731613 10.1016/j.athoracsur.2013.03.094

[27] Duprey A , Trabelsi O, Vola M, Favre JP, Avril S. Biaxial rupture properties of ascending thoracic aortic aneurysms. Acta Biomater 2016;42:273–85. 27345137 10.1016/j.actbio.2016.06.028

[28] Sokolis DP , Kritharis EP, Iliopoulos DC. Effect of layer heterogeneity on the biomechanical properties of ascending thoracic aortic aneurysms. Med Biol Eng Comput 2012;50:1227–37. 22926448 10.1007/s11517-012-0949-x

[29] García-Herrera CM , Celentano DJ, Cruchaga MA, Rojo FJ, Atienza JM, Guinea GV et al Mechanical characterisation of the human thoracic descending aorta: experiments and modelling. Comput Methods Biomech Biomed Eng 2012;15:185–93. 10.1080/10255842.2010.520704 21480018

[30] Olchanyi MD , Sadikov A, Frattolin J, Sasidharan S, Yousuf Salmasi M, Edgar LT et al Validation of markerless strain-field optical tracking approach for soft tissue mechanical assessment. J Biomech 2021;116:110196. 33422728 10.1016/j.jbiomech.2020.110196

