# Supplementary material for: Regional variation in biomechanical properties of ascending thoracic aortic aneurysms
Source: Eur J Cardiothorac Surg. 2022 Jul 27;62(3):ezac392. doi: 10.1093/ejcts/ezac392 (PMC9731372; doi:10.1093/ejcts/ezac392)
Supplement: ezac392_Supplementary_Data [file ezac392_supplementary_data.docx]

**Appendix 1**

Summary of clinical covariates of patients enrolled in the study

| **Covariate** | **Mean(SD) or**  **N /34 (%)** |  | **Covariate** | **Mean(SD or**  **N /34 (%)** |
| --- | --- | --- | --- | --- |
| Age | 61.8 (12.5) |  | Weight (kg) | 81.4 (20.3) |
| Female | 9 (26%) |  | Body mass index (kg/m^2^) | 27.1 (5.4) |
| Height (cm) | 173.1 (10.3) |  | Body surface area (m^2^) | 1.99 (0.28) |
| Max aneurysm diameter (mm) | 54.9 (7.2) |  |  |  |
|  |  |  |  |  |
| Hypertension | 16 (46%) |  | Severe aortic regurgitation | 12 (35.2%) |
| Diabetes | 0 |  | Severe aortic stenosis | 2 (5.9%) |
| Smoking (current or ex) | 11 (32%) |  | Left ventricular ejection fraction | 58.4 (10.4) |

**Appendix 2**

## Anatomical regions and nomenclature of excised ATAA specimen

As the aorta progresses from the sinus to the tubular aorta, its arc of curvature superiorly, posteriorly and eventually to the patient’s left (as the descending aorta) is well-recognised. However, due to geometrical variations of the ascending aorta between subjects, particularly with non-specific distortions associated with aneurysmal disease, the development of an anatomical frames of reference can be challenging. Even the location of fixed landmarks, such as the coronary ostia (proximally) or innominate artery (distally) can vary between subjects. For this study, we chose the fixed landmark being the anterior-most midline of the ascending aorta, at which point the excised specimen was the widest in the vertical dimension (this gives the characteristic “butterfly” appearance when laid out flat (Figure 3)).

Our protocol divides the aneurysm into 3 regions to the anatomical right of the midline (R1, R2, R3) and 3 regions to the left of the midline (L1, L2, L3). Dividing the aneurysm circumference into six regions increases the sensitivity for detecting regional differences in material properties, relative to four regions as previously done(13). The regions for inner curve, outer curve and posterior midline can be estimated with less accuracy compared to regions of equally divided length. R1 and R2, just right of the midline, approximates to the outer curvature of the aorta (which has been described in previous similar studies as the right lateral region (4)), whereas L1 and L2 approximate to the anterior surface. The narrowest portion of the cylindrical structure (centre of the butterfly) corresponds to both the posterior (R3) and inner curve (L3) of the aorta. Note that the upward and lateral curvature of the aorta accounts for the larger ascribed surfaces to both the anterior and right lateral regions.

**Appendix 3: Histological analysis**

**Histological processing**

Whole circumferential strips (~4mm) from the inferior border of the TAA tissue specimen were fixed in 4% Neutral Buffered Formalin, at least 20 times the volume for each sample. This was then allowed to fix for a minimum of 24 hours prior to processing. Each tissue strip was coiled and transferred to individual labelled cassettes. This process was unique and allowed for whole TAA circumferences to be contained in a single slide.

The cassettes were processed on the Leica tissue processor ASP6025 overnight. Tissue samples were then embedded in molten paraffin wax and cooled to a solid medium. 3-4 sections were then taken from each block using the Thermo Scientific Finesse 325 rotary microtome at 3µm. Slides with the section were baked in a 60◦C oven for at least 1 hour or overnight in a 37◦C oven. The following stains were then performed on each section: Haematoxylin & Eosin (H&E), Picrosirius Red, Millers Elastic Van Gieson.

### Computational pathology

Whole slide imaging of full circumferential rings of TAA samples were obtained using a high-resolution digital optical system (Hamamatsu TM) and uploaded onto a digital processing software (QuPath https://qupath.github.io) for further analysis in ImageJ (National Institutes of Health, Laboratory for Optical and Computational Instrumentation, LOCI, University of Wisconsin)(14). Images of each TAA were divided into six equal circumferential regions, matching the regions for mechanical testing (L1, L2, L3, R1, R2, R3). Using a pre-defined workflow, microstructural density calculation of medial structural proteins was conducted, namely of elastin (from slides stained with Elastin Van Gieson) and collagen (slides stained with picrosirius red) (see figure below, A and B). Using the H&E-stained slides, the thresholding function was used to highlight the stained cells of the medial layer. Setting the range of particle size from 20 to 100 µm the total cell count was extracted and divided by the total area to obtain the density (see figure below, C).


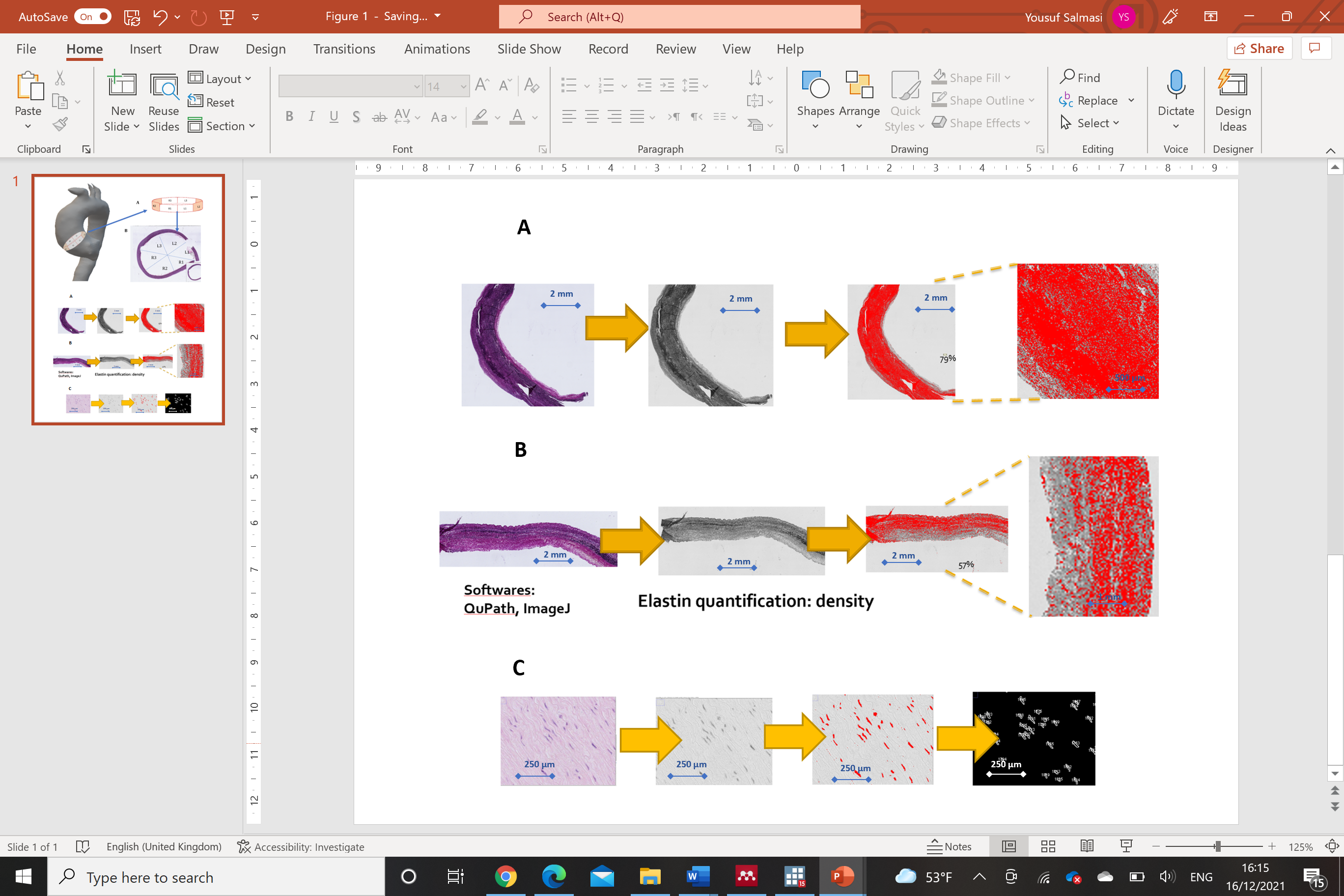


**Appendix 4: calculation for peel force (F peel) and dissection energy**

To quantify the peeling response, the critical energy release rate($G_{c}$), in J m^2^, was calculated, which is defined as(14,15):

$$G_{c}=\frac{W_{ext}-W_{elastic}}{L}$$

Where $W_{ext}$ is the externally applied work, $W_{elastic}$ is the stored energy per unit width, and L is the initial length of the undissected tissue.

$W_{ext}$ is equal to

$$W_{ext}=2Fl$$

where *F* is the mean peeling force, and *l* is the length of the stretched immediately prior to breaking.

$W_{elastic}$ is defined by following equation:

$$W_{elastic}=F(l-L)$$

**Appendix 5**

Paired sample t-tests comparing regional differences in thickness. Table displays p values

| Circumferential region | R2 | R1 | L1 | L2 | L3 |
| --- | --- | --- | --- | --- | --- |
| R3 | 0.0521 | **0.0490** | **0.0066** | **0.0377** | 0.5737 |
| R2 |  | 0.8225 | 0.9767 | 0.4243 | 0.0569 |
| R1 |  |  | 0.7791 | 0.7124 | 0.0706 |
| L1 |  |  |  | 0.1110 | **0.0005** |
| L2 |  |  |  |  | **0.0329** |

**Appendix 6**

Average values for ultimate tensile strength (MPa), based on anatomical region and tissue orientation (circumferential vs longitudinal). Data presented as mean (SD).

Values are given as mean (standard deviation

|  | **Inner curve Anterior Midline Outer curve Posterior** | | | | | |  |
| --- | --- | --- | --- | --- | --- | --- | --- |
|  | L3 | L2 | L1 | R1 | R2 | R3 | |
| Circumferential | 0.786 (0.61) | 0.769 (0.56) | 0.816 (0.69) | 0.867 (0.54) | 0.917 (0.61) | 0.920 (0.57) | |
|  |  |  |  |  |  |  | |
|  |  | | |  | | |  |
|  | L3 | L2 | L1 | R1 | R2 | R3 | |
| Longitudinal | 0.398 (0.28) | 0.494 (0.31) | 0.502 (0.25) | 0.569 (0.31) | 0.662 (0.35) | 0.558 (0.48) | |

**Appendix 7**

Results of ANOVA testing the variation of the two main mechanical properties between circumferential versus longitudinally orientated samples, and between samples from the inner versus the outer curve

| **Source** | **Partial sum of squares** | **Degrees of freedom** | **Mean square** | **F statistic** | **P value** |
| --- | --- | --- | --- | --- | --- |
| **Ultimate tensile strength** | | | | | |
| Model | 3.217 | 2 | 1.609 | 7.88 | 0.0007 |
| Circ. vs long. orientation | 3.185 | 1 | 3.185 | 15.60 | 0.0001 |
| Inner vs outer curve | 0.022 | 1 | 0.022 | 0.11 | 0.741 |
| Residual | 20.011 | 98 | 0.204 |  |  |
| **Peel force** | | | | | |
| Model | 4290.6411 | 2 | 2145.3205 | 3.85 | 0.0227 |
| Circ. vs long. orientation | 4265.391 | 1 | 4265.391 | 7.66 | 0.0061 |
| Inner vs outer curve | 24.144364 | 1 | 24.144364 | 0.04 | 0.8352 |
| Residual | 116906.04 | 210 | 556.69542 |  |  |

**Appendix 8**

| Ultimate tensile strength results: comparison of averaged circumferential vs longitudinal values for each patient  Results of the linear regression fit to this data yields a result of: Coef 1.127, 95% CI 0.24 – 2.01, p=0.014. |
| --- |

**Appendix 9**

Constitutive material parameters of Delfino material model resulting from the fitting of experimental stress-strain response. Data presented as mean (SD).

| Orientation | Segment | ɑ (MPa) | β | RMSE | R^2^ |
| --- | --- | --- | --- | --- | --- |
| Circumferential | L1 | 0.036 (0.02) | 5.438 (4.10) | 0.079 – 0.287 | 0.981 – 0.997 |
|  | L2 | 0.041(0.03) | 5.388 (4.71) | 0.055 – 0.276 | 0.975 – 0.998 |
|  | L3 | 0.035 (0.02) | 5.391 (1.88) | 0.077 – 0.204 | 0.970 – 0.996 |
|  | R1 | 0.046 (0.05) | 5.526 (4.37) | 0.057 – 0.217 | 0.979 – 0.996 |
|  | R2 | 0.063 (0.09) | 5.121 (3.28) | 0.052 – 0.189 | 0.968 – 0.998 |
|  | R3 | 0.036 (0.02) | 4.825 (3.67) | 0.086 – 0.196 | 0.978 – 0.995 |
|  |  |  |  |  |  |
| Longitudinal | L1 | 0.048 (0.08) | 4.660 (2.71) | 0.057 – 0.200 | 0.975 – 0.997 |
|  | L2 | 0.035 (0.03) | 5.423 (1.77) | 0.081 – 0.206 | 0.992 – 0.994 |
|  | L3 | 0.039 (0.03) | 4.842 (1.95) | 0.088 – 0.191 | 0.988 – 0.993 |
|  | R1 | 0.047 (0.06) | 3.969 (2.61) | 0.069 – 0.184 | 0.961 – 0.995 |
|  | R2 | 0.049 (0.04) | 4.070 (2.47) | 0.088 – 0.157 | 0.977 – 0.993 |
|  | R3 | 0.036 (0.03) | 5.054 (2.83) | 0.091 – 0.202 | 0.993 – 0.990 |

Constitutive material parameters of Ogden material model. Results from the fitting of experimental stress-strain response. Data presented as mean (SD).

| Orientation | Segment | µ1 (MPa) | ɑ1 | µ2 (MPa) | ɑ2 | RMSE | R^2^ |
| --- | --- | --- | --- | --- | --- | --- | --- |
| Circumferential | L1 | 1.272 (3.09) | 4.703 (5.66) | 0.453 (1.27) | 5.411 (5.75) | 0.029 – 0.228 | 0.975 – 0.999 |
|  | L2 | 2.750 (5.36) | 5.153 (5.69) | 0.275 (1.21) | 5.447 (6.21) | 0.031 – 0.129 | 0.985 – 0.999 |
|  | L3 | 1.651 (3.58) | 4.284 (5.45) | 0.570 (0.82) | 3.696 (4.68) | 0.021 – 0.143 | 0.983 – 0.999 |
|  | R1 | 0.751 (1.28) | 5.059 (5.80) | 0.010 (0.55) | 4.821 (4.59) | 0.028 – 0.145 | 0.980 – 0.999 |
|  | R2 | 0.384 (0.89) | 7.550 (5.65) | -0.301 (2.24) | 2.059 (3.82) | 0.030 – 0.164 | 0.975 – 0.999 |
|  | R3 | 1.781 (2.91) | 4.512 (5.05) | 0.260 (1.94) | 4.205 (5.21) | 0.037 – 0.127 | 0.988 – 0.998 |
|  |  |  |  |  |  |  |  |
| Longitudinal | L1 | 1.842 (3.46) | 1.451 (4.59) | 0.442 (1.11) | 5.612 (6.22) | 0.021 – 0.097 | 0.991 – 0.999 |
|  | L2 | 1.383 (2.20) | 4.387 (7.55) | -0.173 (0.98) | 3.585 (4.44) | 0.023 – 0.115 | 0.991 – 0.999 |
|  | L3 | 1.022 (1.06) | 3.085 (4.49) | -0.036 (0.85) | 5.322 (3.94) | 0.030 – 0.108 | 0.989 – 0.999 |
|  | R1 | 0.804 (1.35) | 2.429 (4.96) | -0.872 (1.99) | 4.994 (4.36) | 0.018 – 0.179 | 0.972 – 0.999 |
|  | R2 | 0.475 (0.06) | 0.477 (7.53) | 0.372 (1.13) | 5.195 (3.33) | 0.031 – 0.998 | 0.039 – 0.999 |
|  | R3 | 0.467 (0.48) | 0.508 (3.74) | 0.573 (0.86) | 5.212 (5.27) | 0.036 – 0.080 | 0.993 – 0.999 |

**Appendix 10**

Average values for delamination data, based on anatomical region and tissue orientation (circumferential vs longitudinal). Data presented as mean (SD).

Values are given as mean (standard deviation

|  | **Anterior** | **Inner Curve** | **Outer Curve** | **Posterior** |
| --- | --- | --- | --- | --- |
| Peel force (Nm/mm) | | | | |
| Circumferential | 29.9 (15) | 26.8 (15) | 32.5 (14) | 31.3 (19) |
| Longitudinal | 38.5 (34) | 43.8 (40) | 35.5 (22) | 39.4 (21) |
| Dissection energy (J m^2^) | | | | |
| Circumferential | 72.2 (41) | 91.4 (108) | 79.8 (39) | 81.2 (60) |
| Longitudinal | 91.1 (68) | 115.7 (88) | 88.5 (69) | 103.3 (69) |

**Appendix 11**

Linear regression analysis: association between patient covariates and TAA material properties (wall thickness, tensile/peel properties)

| **Covariate** | **Coef** | **Standard error** | **95% CI** | **P value** |
| --- | --- | --- | --- | --- |
| **Tissue thickness** | | | | |
| Patient age | 0.0125 | 0.004 | 0.0044 - 0.0207 | **0.004** |
| Max aneurysm diameter | 0.0152 | 0.010 | -0.0056 – 0.0361 | 0.139 |
| Pulse wave velocity | 0.1728 | 0.050 | 0.0595 - 0.2861 | **0.007** |
| Smoking | 0.2022 | 0.138 | -0.0793 - 0.4837 | 0.153 |
| **Ultimate tensile strength - circumferential** | | | | |
| Patient age | -0.0044 | 0.0108 | -0.0287 – 0.0200 | **<0.001** |
| Max aneurysm diameter | 0.0092 | 0.014 | -0.0272 – 0.0456 | 0.544 |
| Pulse wave velocity | -0.0766 | 0.0442 | -0.2172 – 0.0641 | 0.182 |
| Smoking | 0.0217 | 0.2079 | -0.4041 - 0.4477 | 0.917 |
| **Ultimate tensile strength - longitudinal** | | | | |
| Patient age | -0.0097 | 0.003 | -0.0160 - -0.0035 | **0.004** |
| Max aneurysm diameter | -0.0141 | 0.008 | -0.0315 - 0.0034 | 0.105 |
| Pulse wave velocity | -0.0761 | 0.026 | -0.1353 - -0.0168 | **0.017** |
| Smoking | 0.0671 | 0.094 | -0.127 - 0.261 | 0.484 |
| **Peel force - circumferential** | | | | |
| Patient age | -0.660 | 0.173 | -1.016 - -0.305 | **0.001** |
| Max aneurysm diameter | -0.568 | 0.481 | -1.607 – 0.472 | 0.259 |
| Pulse wave velocity | -1.978 | 1.771 | -5.985 – 2.028 | 0.293 |
| Smoking | 2.016 | 5.100 | -8.430 - 12.463 | 0.696 |
| **Peel force - longitudinal** | | | | |
| Patient age | -0.404 | 0.443 | -1.318 – 0.510 | 0.371 |
| Max aneurysm diameter | -0.506 | 1.190 | -3.077 – 2.065 | 0.678 |
| Pulse wave velocity | -1.189 | 7.298 | -17.699 – 15.321 | 0.874 |
| Smoking | 13.160 | 11.152 | -9.764 - 36.084 | 0.249 |
| **Dissection energy - circumferential** | | | | |
| Patient age | -1.642 | 0.669 | -3.022 - -0.262 | **0.022** |
| Max aneurysm diameter | -1.948 | 1.491 | -5.170 – 1.274 | 0.214 |
| Pulse wave velocity | -4.648 | 4.846 | -15.612 – 6.315 | 0.363 |
| Smoking | 1.885 | 18.868 | -36.899 -40.669 | 0.921 |
| **Dissection energy - longitudinal** | | | | |
| Patient age | -1.721 | 1.059 | -3.906 – 0.465 | 0.117 |
| Max aneurysm diameter | -1.594 | 2.782 | -7.604 – 4.415 | 0.576 |
| Pulse wave velocity | -6.953 | 14.874 | -40.601 – 26.695 | 0.651 |
| Smoking | 19.729 | 28.062 | -37.955 - 77.413 | 0.488 |

*Both tensile and peel parameters were measured in two directions – circumferential, longitudinal. Bold indicates statistical significance.*

**Appendix 12**

Linear regression analysis: association between other patient covariates and TAA wall thickness, UTS and peel force

BMI = body mass index; LVEF = left ventricular ejection fraction; MAP = mean arterial pressure; PWV = pulse wave velocity. Bold indicates statistical significance.

| **Covariate** | **Coef** | **Standard error** | **95% CI** | **P value** |
| --- | --- | --- | --- | --- |
| **Tissue thickness** | | | | |
| Patient height | 0.0043 | 0.005 | -0.0068 - 0.0155 | 0.412 |
| Patient BMI | -0.0063 | 0.012 | -0.0319 - 0.0192 | 0.596 |
| LVEF | 0.00015 | 0.006 | -0.0126 - 0.0129 | 0.980 |
| MAP | -0.0038 | 0.003 | -0.0112 - 0.0035 | 0.280 |
| **Circumferential UTS** | | | | |
| Patient height | 0.00274 | 0.0057 | -0.0102 – 0.0156 | 0.642 |
| Patient BMI | 0.0014 | 0.0122 | -0.0263 - 0.0291 | 0.911 |
| LVEF | -0.00068 | 0.0087 | -0.0218 – 0.0205 | 0.940 |
| MAP | 0.0035 | 0.0034 | -0.0041 – 0.0112 | 0.324 |
| **Longitudinal UTS** | | | | |
| Patient height | 0.0001 | 0.004 | -0.0085 – 0.0099 | 0.875 |
| Patient BMI | -0.0050 | 0.007 | -0.0203 - 0.0103 | 0.506 |
| LVEF | -0.0010 | 0.005 | -0.0108 - 0.0089 | 0.840 |
| MAP | 0.0014 | 0.003 | -0.0050 – 0.0079 | 0.651 |
| **Circumferential Peel force** | | | | |
| Patient height | 0.152 | 0.241 | -0.345 – 0.648 | 0.535 |
| Patient BMI | 0.016 | 0.437 | -0.884 – 0.915 | 0.972 |
| LVEF | -0.010 | 0.233 | -0.500 – 0.479 | 0.965 |
| MAP | 0.067 | 0.142 | -0.225 – 0.360 | 0.639 |
| **Longitudinal Peel force** | | | | |
| Patient height | 0.044 | 0.539 | -1.069 – 1.156 | 0.936 |
| Patient BMI | -1.215 | 0.882 | -3.036 – 0.605 | 0.181 |
| LVEF | -0.380 | 0.614 | -1.676 – 0.915 | 0.544 |
| MAP | -0.148 | 0.368 | -0.909 – 0.613 | 0.692 |
| **Circumferential dissection energy** | | | | |
| Patient height | 0.448 | 0.890 | -1.391 – 2.286 | 0.620 |
| Patient BMI | -1.422 | 1.494 | -4.505 – 1.661 | 0.351 |
| LVEF | 0.284 | 0.905 | -1.626 – 2.193 | 0.758 |
| MAP | 0.469 | 0.595 | -0.761 – 1.698 | 0.439 |
| **Longitudinal dissection energy** | | | | |
| Patient height | 0.308 | 1.334 | -2.445 – 3.062 | 0.819 |
| Patient BMI | -2.682 | 2.203 | -7.228 – 1.865 | 0.235 |
| LVEF | -0.700 | 1.484 | -3.830 – 2.431 | 0.643 |
| MAP | -0.372 | 0.901 | -2.236 – 1.493 | 0.684 |

**Appendix 13**

| **Histological feature** | **Coef** | **Standard error** | **95% CI** | **P value** |
| --- | --- | --- | --- | --- |
| **Circumferential UTS** | | | | |
| Elastin | 0.0122 | 0.007 | -0.0012 - 0.0257 | 0.072 |
| Collagen | 0.0515 | 0.016 | 0.0188 - 0.0841 | **0.004** |
| VSMC count | -0.0004 | 0.005 | -0.0119 – 0.0110 | 0.932 |
| **Longitudinal UTS** | | | | |
| Elastin | 0.0034 | 0.003 | -0.0022 - 0.0091 | 0.225 |
| Collagen | 0.0258 | 0.006 | 0.0124 – 0.0392 | **0.001** |
| VSMC count | -0.0020 | 0.002 | -0.0070 – 0.0031 | 0.416 |
| **Circumferential peel force** | | | | |
| Elastin | 0.284 | 0.161 | -0.048 – 0.616 | 0.090 |
| Collagen | 1.161 | 0.373 | 0.372 – 1.950 | **0.006** |
| VSMC count | -0.156 | 0.124 | -0.414 – 0.102 | 0.223 |
| **Longitudinal peel force** | | | | |
| Elastin | 0.084 | 0.353 | -0.644 – 0.812 | 0.814 |
| Collagen | 1.044 | 0.521 | -0.061 – 2.149 | 0.062 |
| VSMC count | 0.187 | 0.288 | -0.411 – 0.786 | 0.522 |
| **Circumferential DEF** | | | | |
| Elastin | 0.646 | 0.571 | -0.533 – 1.825 | 0.269 |
| Collagen | 3.663 | 1.184 | 1.152 – 6.174 | **0.007** |
| VSMC count | 0.182 | 0.716 | -1.307 – 1.672 | 0.801 |
| **Longitudinal DEF** | | | | |
| Elastin | 0.735 | 0.862 | -1.042 – 2.514 | 0.401 |
| Collagen | 3.975 | 1.670 | 0.434 – 7.516 | **0.030** |
| VSMC count | 0.183 | 0.716 | -1.307 – 1.672 | 0.801 |
